# Coping strategies among mothers of children with leukemia in Tunisia

**DOI:** 10.1192/j.eurpsy.2022.1693

**Published:** 2022-09-01

**Authors:** S. Dhakouani, M. Karoui, S. Jammeli, R. Kammoun, F. Ellouz

**Affiliations:** 1 Razi hospital, G, Tunis, Tunisia; 2 Razi Hospital, Psychiatry G, manouba, Tunisia

**Keywords:** coping, psychooncology, leukemia, mothers

## Abstract

**Introduction:**

The diagnosis of leukemia in a child is traumatic life experience that negatively affects parents and especialy the mother which is the “caregiver” who assists and coordinates all stages of treatment.

**Objectives:**

To determine the prevalence of psychological distress among mothers of tunisian children with leukemia and to investigate their coping strategies.

**Methods:**

A cross-sectional study was conducted at Aziza Othmana hospital department of pedo-oncology in Tunisia between June and July 2021. HADS scale was used to estimate the prevalence of anxiety and depression and coping strategies were measured via arabic version of the brief cope scale.

**Results:**

We included 31 mothers, their middle age was 41 years old. In this study we didn’t include mothers with psychiatric history. Acute lymphoblastic leukemia was the most frequent type of cancer in our sample (94%). The middle age of the children was 10 years old and all of them were under chemotherapy. Clinically significant levels of anxiety and depression were reported by 58% and 49% of mothers, respectively. In our study, 81% of the participants practiced prayer and all mothers turned to religion as a coping strategy. Approach coping styles (especially acceptance and planning) were more frequently used than avoidant coping styles (especially substance use and denial).

**Conclusions:**

Mothers are profoundly affected by a child’s cancer diagnosis, they should have early assessment of their mental health needs to have access to appropriate interventions.

**Disclosure:**

No significant relationships.

